# Knowledge translation concerns for the CONSORT-PRO extension reporting guidance: a review of reviews

**DOI:** 10.1007/s11136-022-03119-w

**Published:** 2022-03-26

**Authors:** Rebecca Mercieca-Bebber, Olalekan Lee Aiyegbusi, Madeleine T. King, Michael Brundage, Claire Snyder, Melanie Calvert

**Affiliations:** 1 NHMRC Clinical Trials Centre, Faculty of Medicine and Health, University of Sydney, Sydney, Australia; 2 Centre for Patient Reported Outcomes Research, and Birmingham Health Partners Centre for Regulatory Science and Innovation, Institute of Applied Health Research, University of Birmingham, and University Hospitals Birmingham NHS Foundation Trust, Birmingham, UK; 3 National Institute for Health Research (NIHR) Birmingham Biomedical Research Centre, NIHR Surgical Reconstruction and Microbiology Research Centre, NIHR Applied Research Centre West Midlands, University of Birmingham, Birmingham, UK; 4 School of Psychology, Faculty of Science, University of Sydney, Sydney, Australia; 5 Cancer Care and Epidemiology, Department of Oncology, Cancer Research Institute, Queen’s University, Kingston, ON Canada; 6 Johns Hopkins School of Medicine and Bloomberg School of Public Health, Baltimore, MD USA

**Keywords:** Reporting, Research waste, CONSORT-PRO, Patient-reported outcomes, Quality of life, Research methodology

## Abstract

This review of reviews aimed to appraise the use of the CONSORT-PRO Extension as an evaluation tool for assessing the reporting of patient-reported outcome (PROs) in publications, and to describe the reporting of PRO research across reviews. We also outlined how variation in such evaluations impacts knowledge translation and may lead to potential misuse of the CONSORT-PRO Extension. We systematically searched Medline, Pubmed and CINAHL from 2013 to 2025 March 2021 for reviews of the completeness of reporting of PRO endpoints according to CONSORT-PRO criteria. Two reviewers extracted details of each review, the percentage of included studies that addressed each CONSORT-PRO item, and key recommendations from each review. Fourteen reviews met inclusion criteria, and only six of these used the full CONSORT-PRO checklist with minimal justified modifications. The remaining eight studies made significant or unjustified adjustments to the CONSORT-PRO Extension. Review studies also varied in how they scored multi-component CONSORT-PRO items. CONSORT-PRO items were often unreported in trial reports, and certain CONSORT-PRO items were reported less often than others. The reporting of statistical approaches to dealing with missing PRO data were poor in RCTs included in all 14 review articles. Studies reviewing PRO publications often omitted recommended CONSORT-PRO items from their evaluations, which may cause confusion among readers regarding how best to report their PRO research according to the CONSORT-PRO extension. Many trials published since CONSORT-PRO’s release did not report recommended CONSORT-PRO items, which may lead to misinterpretation and consequently to research waste.

**Supplementary Information:**

The online version contains supplementary material available at 10.1007/s11136-022-03119-w.

## Background

Incomplete, unclear or poor quality reporting of patient-reported outcome (PRO) endpoints in randomised controlled trials (RCTs) is a well-documented problem [1, 2], which can lead to research waste in two key ways. Firstly, unclear reporting may lead to inaccurate messages, misinterpretation or misunderstanding of the aims, methods, findings, or relevance of the data. In turn, this reduces the ability of policy makers, funders, clinicians and patients to use the PRO data for clinical and policy decision-making [3–5]. Secondly, poorly communicated PRO research may lead others to assume that certain research questions have not been answered [4]. This could lead others to repeatedly or unnecessarily reproduce the research and spend additional, scarce research funding and time to answer the same questions., [3]. Similarly, failing to report certain findings will most certainly lead others to believe that research questions have not been answered. This applies to trials for which PROs have not been reported at all, or where certain measured PRO domains from multi-dimensional measures have not been reported. Non-reporting is particularly concerning, given that past studies have estimated that PROs are left completely unreported for 38–80% trials[6, 7]. In one of these reviews, PRO data were left unreported for 49,568 participants across 61 trials and multiple time points [6], which is unethical, as it devalues the participation of patients in clinical trials and prevents valuable data from being used to inform future patient care.

Reporting guidelines are simple, structured tools for health researchers to use while writing manuscripts. They provide a minimum list of information needed to ensure a manuscript can be understood by readers, used for clinical decision-making, included in systematic reviews, and to ensure the research is reproducible [5, 8]. Evidence of sub-optimal PRO reporting [1, 2, 9, 10] led to the 2013 release of the CONsolidated Standards Of Reporting Trials (CONSORT) PRO extension [11, 12] reporting guidelines. CONSORT-PRO aims to promote transparent reporting of RCTs in which PROs are primary or important secondary outcomes. If used appropriately it can help reduce waste due to incomplete or unclear PRO reporting, and it can best achieve this goal if used in its entirety. Its use is endorsed by many stakeholders, including Enhancing the QUAlity and Transparency of health Research (EQUATOR) [13] and the European Medicine’s Agency [14]. The CONSORT-PRO Extension includes 14 items: five PRO-specific extension items and nine PRO-specific elaborations to CONSORT-2010 items. Addressing all 14 items promotes complete reporting of clinical trial PRO endpoints. Whilst adherence to the CONSORT-PRO Extension does not imply the study was designed and conducted according to highest research standards, adherence will allow such value judgements to be made by the reader.

Since the 2013 publication of CONSORT-PRO, several studies have reviewed PRO research publications to assess the extent to which these reports address the CONSORT-PRO criteria (e.g., [6, 15–17]). However, the appraisal checklists used in these review articles have varied. Some have used a 14-item checklist (one item per assessment point), while others have split certain CONSORT-PRO items into multiple items, acknowledging that certain checklist items address multiple reporting requirements. Some reviews have limited their appraisals to only the five PRO-specific extension items. Not including the nine PRO-specific CONSORT-2010 elaborations when assessing completeness of PRO reporting may cause readers to misinterpret what constitutes comprehensive reporting and could perpetuate poor reporting practices. In turn, incomplete reporting can lead to waste as described above, and may have implications for knowledge translation. Knowledge translation refers to the “synthesis, exchange, and application of knowledge by relevant stakeholders to accelerate the benefits of global and local innovation in strengthening health systems and improving people’s health” [18] p.9.

Although it must be noted that the CONSORT-PRO guidance was never intended to be used as an evaluation tool, it continues to be used in this way. Review studies to date have either explicitly stated or implied that a high score indicates high-quality reporting. However, if CONSORT-PRO items are left out of such evaluations, there is a risk that readers may be misled about how to write their future PRO articles completely and transparently—which is a knowledge translation concern.

We aimed to appraise the use of the CONSORT-PRO Extension in “review articles” that have assessed the quality of reporting in PRO research publications and to describe the reporting of PRO research across reviews. We also discuss how this impacts knowledge translation and potential misuse of the CONSORT-PRO Extension.

## Methods

Our review methodology followed PRISMA guidelines [19].

### Search strategy

Medline, Pubmed and CINAHL were systematically searched from 2013 (year of CONSORT-PRO publication) until 25 March 2021 using the following terms: ((((((((CONSORT-PRO) OR ((CONSORT adj3 patient-reported outcome))) OR ((CONSORT adj3 PRO))) OR ((reporting quality adj3 PRO))) OR ((reporting quality adj3 patient-reported outcome*))) OR ((reporting complet* adj3 patient-reported outcome*))) OR ((reporting complet* adj3 PRO))) OR (reporting quality adj3 PROs)) OR (reporting complet* adj3 PROs). These databases were chosen for their relevance and because key papers known to the authors were indexed in these databases. Duplicate references were removed in Endnote X9.

### Selection criteria

Articles reviewing the completeness of reporting of PRO endpoints in RCTs using CONSORT-PRO criteria were included. Articles that reviewed the completeness of PRO reporting using other checklists were excluded. Abstracts were double screened using these criteria by two authors (RMB, OLA). Full text articles were reviewed by one author (RMB) and checked against criteria by OLA. All discrepancies were resolved through discussion.

### Data extraction

Two authors (RMB, OLA) extracted the data described in Box 1 from included articles. Data were summarised using descriptive statistics and narrative synthesis.

Box 1 Information extracted from review articles About the review article

- Study aim.
- Date window for the search.
- Did the review article include papers published before Feb 2013 (CONSORT-PRO publication)?
- Number of study publications reviewed.
- Disease area/s of included papers.
- Study selection criteria.
- Terminology/definition of what the review article claimed to assess using CONSORT-PRO. Where multiple terms were used, we extracted the term described in the Methods, or failing that, the Results tables.

The CONSORT-PRO checklist used

- The checklist used to review the included studies.
- Any CONSORT-PRO items that were missing from the scoring sheet/evaluation.
- Specific instructions that reviewers were given to assess the included studies.
- How were the included studies scored, e.g., by checklist item, total score, both?
- If a total score was used, what was the best possible total score?
- Key concerns with the checklist used (if any), i.e., whether any items from the full checklist were omitted, were items weighted if a total score was used, etc.
- Did the review article authors justify their modifications to the checklist for evaluation purposes?
- Did the review article authors review protocols of the included studies using SPIRIT-PRO?
- Did the review article mention SPIRIT-PRO [20] at any point?

Findings of the article

- Summary of the results for individual CONSORT-PRO items; i.e., what items scored best or worst?
- Percentage of studies addressing each checklist item.
- Summary of CONSORT-PRO total scores.
- Other comments on results.
- Conclusions from the article.

Recommendations by the article

- Recommendations given by the review article for how to improve reporting or reduce waste due to poor reporting.
- Did the review article make any judgements about what was a “good” CONSORT-PRO score?

The journal

- Did the publishing journal include a recommendation to use CONSORT-PRO in their author instructions? [17].

## Results

**Fig. 1:**
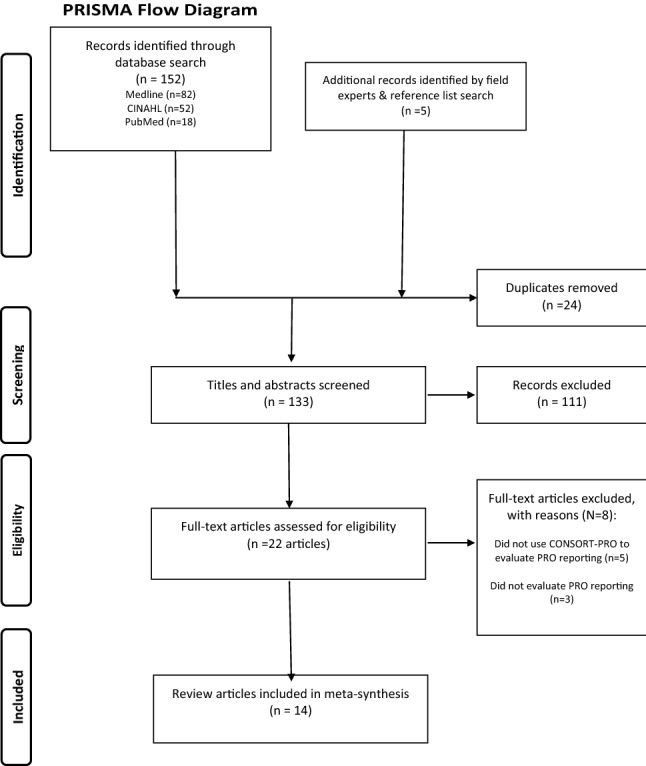
PRISMA flow diagram

Our database searches retrieved 152 articles, of which 14 met criteria for inclusion in our review (Fig. 1), listed in Appendix 1. The 14 review articles were published between 2015 and 2020, including a total of 1186 trials published between 1998 and 2019. The most common disease area studied by the review articles was oncology, *n* = 8 (Table 1). Two review articles also reviewed protocols of the included studies using SPIRIT-PRO.

**Table 1:** Characteristics of the 14 included review studies

Characteristic	Number of studies (*N* = 14)
*Year of publication*	
2015	2
2016	3
2017	3
2018	1
2019	3
2020	2
*Disease or condition of focus*	
Oncology	8
Haematological malignancies	1
Cardiology	1
General surgery	1
Osteoporosis	1
Tendinopathy	1
Various	1
*Authors’ description of what was assessed using CONSORT-PRO criteria*	
Adherence to CONSORT-PRO	5
Reporting quality	3
Methodological quality	2
Completeness	2
PRO reporting	1
Reporting standards	1
*CONSORT-PRO criteria used to review studies*	
Full checklist with all criteria, minimal (justified) to no modifications	6
Modified checklist with few criteria excluded, however excluded criteria were extension items	1
Modified checklist with 3 or more criteria excluded	4
Five extension items only	3
*Scoring instructions for assessing papers using the CONSORT-PRO criteria*	
Scoring instructions detailed	9
Scoring instructions omitted or unclear	5
*How CONSORT-PRO scoring was reported*	
Individual checklist item scores only	5
Total score only	0
Checklist items and total score	9
*Did the review article make any judgements about what was a "good" overall CONSORT-PRO score?*	
No	13
Yes	1

### Variation in use of CONSORT-PRO as an evaluation tool

Although all studies used the CONSORT-PRO checklist to evaluate reporting of PROs, the definition of what was being evaluated in each review differed (Table 1). Definitions of what was assessed included “adherence to CONSORT-PRO criteria” *n* = 5 [16, 17, 21–23], “reporting quality” *n* = 3 [15, 24, 25], “methodological quality” *n* = 2 [26, 27], “reporting completeness” *n* = 2 [6, 28], “PRO reporting” *n* = 1 [29], and “reporting standards” *n* = 1 [30]. Many of the articles used multiple different terms throughout the manuscript.

Some studies modified CONSORT-PRO criteria for evaluation, and sometimes these changes were not described or justified in the methods, but apparent from the presentation of results or supplementary material. Only six studies used the full CONSORT-PRO checklist without modification or with minimal justified modifications [6, 17, 21, 23, 25, 29]. The remaining eight studies made significant or unjustified adjustments to the CONSORT-PRO Extension: *n* = 5 studies excluded three or more CONSORT-PRO elaboration items without justification, *n* = 3 studies excluded all elaboration items and assessed studies using only the five CONSORT-PRO Extension items.

Two issues impacted comparability of total scores across these review articles: the CONSORT-PRO items evaluated and their weighting in the total score. While most CONSORT-PRO items were awarded a score of 1 each time it was addressed by each RCTs, some CONSORT-PRO items that contained multiple criteria were divided for scoring, as shown in Table 2. For example, item P6a: “evidence of PRO instrument validity and reliability should be provided or cited if available, including the person completing the PRO and methods of data collection (paper, telephone, electronic, other)” was spilt in to two or three criteria for two and eight studies, respectively. When this was done, the overall value of the item was increased. When this was not done, it was not abundantly clear if studies had addressed all three components of the item, or only part of the criteria.

**Table 2:** Adherence to CONSORT-PRO criteria- summary of findings from 14 systematic reviews

Review publication & reporting checklist used	Bylicki et al. (2014)	Chen et al. (2020)	Dos Santos et al. (2017)	Efficace et al. (2015)	Evans et al. (2019)	Kyte et al. (2019)	Le Blanc et al. (2020)^a^	Mack et al. (2018)^a^	Martini et al. (2019)^a^	Mercieca-Bebber, et al. (2017) *QLR*	Mercieca-Bebber et al. (2017) JPRO	Stevens et al. (2016)	Van Der Weijst et al. (2019)	Weingartner et al. (2016)
Publication sample reviewed	124 Phase III medical oncology RCTs published 2007–2011	37 RTCs of cardiac catheter ablation of the heart, published 2000–2019	22 molecular targeted therapy RCTs for metastatic renal cell carcinoma, published 2005–2015	557 RCTs in the PROMOTION registry, published 2004–2014	4 UK RCTs lateral elbow tendinopathy, published 2003–2014	99 cancer RCTs on NIHR UK portfolio 2001–2014, published by 2017	30 studies relapsed/refractory multiple myeloma, pre and post 2013	23 studies of exercise management for osteoporosis published, 1998–2017	48 studies Gastroenteropancreatic neuroendocrine tumours, published 1999–2016	66 RCTs published post CONSORT-PRO; 2013–2015 (case//control)	36 Phase III ovarian cancer RCTs published 2000- Feb 2016	20 general surgery RCTs, published 2007–2012	85 stage IIIB and/or IV NSCLC RCTs, published 2007–2017	30 advanced cancer RCTs, Published 2010-March 2013
P1b. PRO identified in abstract	28%	62%	41%	81%	1/4	32%	73.3%	91.3%	19%	96%//59%	69%	25.7%	60%	30%
*- as primary or secondary outcome*										38%//38%	47%			
(2a) Background and rationale for PRO assessment	43%	43%	41%	N/A	N/A	34%	60%	34.8%	N/A	85%//93%	42%	25.7%	N/A	17%
P2b. The PRO hypothesis should be stated & relevant domains identified, if applicable	26%	8%	4.5%	17%	0/4	28%	10%	39.1% hypothesis; 82.6% hypothesis with domains	15%	73%//23% (including domains: 50%//15%)	19%	14.3%	13%	13%
(4a) Eligibility—if PROs were used in eligibility or stratification criteria	N/A	N/A	N/A	N/A	N/A	2%	N/A	N/A	N/A	0%	0% of eligible	0%	N/A	N/A
P6a. Evidence of PRO instrument validity and reliability should be provided or cited if available,	36%	32%	55%	76%	0/4 in UK population, 2/4 in another population	32%	50%	47.8%	95%	92%//73%	64%	20%	6%	70%
including:*-The person completing the PRO*	38%		32%	24%	N/A		56.7%	60.9%	Instrument administration: 60%	81%//78%	53%	54.3%		
*-methods of data collection (paper, telephone, electronic, other)*	16%		0%		1/4		16.7%	8.7%		35%//25%	6%	42.9%		
(7a) How sample size was determined – not required for PRO unless it is a primary study outcome	N/A	100% of RCTs with primary endpoints (*n* = 1)	N/A	N/A	N/A	63%	N/A	16.7%	N/A	67%//63%	0% of eligible	N/A	N/A	0%
P12a. Statistical approaches for dealing with missing data are explicitly stated	37%	32%	32%	20%	0/4	32%	23.3%	13%	21%	77%//50%	39%	2.9%	18%	27%
(13a) The number of PRO outcome data *-at baseline*	61%	16%	50%	N/A	N/A	27%	40%	91.3%	39%	73%//68%	50%	N/A	N/A	57%
*-and subsequent time points should be made transparent*	70%		50%	N/A	N/A			98.7%	N/A	81%//73%	47%	13.1%	N/A	
(15) A table showing baseline PRO data when collected	40%	89%	32%	N/A	N/A	25%	53.3%	95.7%	N/A	73%//85%	36%	31.4%	N/A	17%
(16) For each group, *-the number of participants (denominator) included in each group analysis*	48%	49%	55%	N/A	N/A	40%	N/A	100%	Missing data reported: 79%	81%//73%	64%	13.1%	N/A	60%
*-and whether the analysis was by original assigned groups—required for PRO results*	N/A			N/A	N/A		N/A		N/A			N/A	N/A	
(17a) For each primary and secondary outcome:- *results for each group*	43%	65%	41%	N/A	N/A	30%	73.3%	91.3%	N/A	92%//85%	72%	N/A	N/A	67%
- *the estimated effect size, and its precision (such as 95% confidence interval)*—for multidimensional PROs results from each domain and time point	N/A			N/A	N/A			34.8%	N/A	81%//75%	44%	N/A	N/A	
(18) Results of any other analyses performed, including subgroup analyses and adjusted analyses, distinguishing pre-specified from exploratory—including PRO analyses, where relevant	N/A	100% (4/4 applicable studies)	N/A	N/A	N/A	12%	N/A	21.7%	N/A	54%//45%	39%	0%	N/A	7%
P20/21. PRO-specific *-limitations*	35%	35%	18%	46%	0/4	24%	26.7%	34.8%	N/A	77%//75%	36%	22.6%	31%	23%
*-and implications for generalizability and clinical practice*								65.2%	PRO results discussed: 64%	81%//83%	53%			
(22) PRO data should be interpreted in relation to clinical outcomes including survival data, where relevant	60%	30%	50%	N/A	N/A	31%	93.3%	82.6%	N/A	81%//85%	69%	74.3%	N/A	53%

Percentages reported indicate the percentage of trials from each review that addressed that criterion; N/A = not assessed; where studies reported results graphically, we approximated the percentage for each item to the best of our ability. Percentages were calculated for Weingartner et al. based on data provided

^a^Study reviewed RCTs as well as other research designs (e.g., observational studies)

Review articles varied in whether they reported CONSORT-PRO item-level scores only [16, 22, 24, 27, 30], or both total scores (i.e., total number of CONSORT-PRO items addressed) and item-level scores [6, 15, 17, 21, 23, 25, 26, 28, 29]. The highest possible total score varied between review articles (range 11–15), even among those that used the full CONSORT-PRO checklist, due to differences in how individual items were weighted. Of the three studies using just the five CONSORT-PRO Extension items, none reported a total score [16, 22, 27].

### Item-level evaluations using the CONSORT-PRO guidelines

The frequency of reporting of some CONSORT-PRO items varied markedly among review articles (Tables 2, 3). For example, identification of the PRO as a primary or secondary endpoint was addressed by more than 60% of RCTs in five reviews [16, 21, 25, 27, 28], but less than 30% of RCTs in three reviews [15, 22, 24]. Statistical approaches to dealing with missing PRO data were poorly reported in RCTs included in all 14 review articles. Across the 14 review articles, the total number of CONSORT-PRO items (as included in each respective evaluation checklist) addressed by RCTs ranged from 0 to 100%.

**Table 3:** Summary of findings of the 14 review articles

Lead author, year	Study aim as stated by the authors	Summary of study’s conclusion	Summary of the results for individual CONSORT-PRO items; i.e., what items scored best or worst, as stated by the authors	Summary of CONSORT-PRO total scores	Recommendations for improving reporting or reducing waste due to poor reporting, as stated by the authors
Bylicki (2015)	The aim was to (1) evaluate the quality of PROs reporting in recent oncology RCT reports, according to the 2013 PROs-specific CONSORT extension, thereby establishing the current adequacy of PROs reporting using the 11-point PROs reporting quality score (PRORQS). (2) investigate manuscripts’ characteristics associated with better quality in PROs reporting	PROs were often poorly reported. The main exception was where PROs were reported in separate PROs-dedicated manuscripts	124 articles were included in the review. Ten manuscripts reported correctly all five extensions of the CONSORT statement (P1b, P2b, P6a, P12a, P20/21). The most frequently reported item included in the 2013 CONSORT extension was the identification of the PROs relevant domains (item P2b, correctly reported in 65% of the RCTs). A correct description of the prespecified PROs hypothesis was reported in 26% of the reports, and the description of the analysis power in 10% of the reports. The identification of the PROs in the abstract as a primary or secondary outcome (item P1b) was done in 28% of the reports. Also, 16% reported methods for PROs data collection (paper, telephone, electronic, other). Statistical approaches for managing missing data (item P12a) were adequately described in 37% of RCTs. Adequate description of each domain result for multidimensional PROs (item E17a) was found in 43% of manuscripts. PROs-specific limitations (item P20/21) were discussed in 35% of manuscripts. Among the 11 items included in the PRORQS, none was correctly reported by more than 70% of RCT reports	The mean PROs reporting quality score (PRORQS) for all items was 5.0 on an 11-point scale [range: 0–11, 95% CI of the mean 4.4–5.5], including 17 publications (14%) having a PRORQS ≤ 1. Four trials (3%) were found with a score of 11. All were reported in a secondary PROs-specific manuscript, and three were pivotal trials with positive results	Although not mentioned in the CONSORT extension guidelines,… oncology RCT (publications) should provide a short report of PROs in the primary manuscript, with a more exhaustive subsequent publication in a dedicated PROs-focused manuscript
Chen (2020)	1. How many RCTs of cardiac catheter ablation (CCA) include an assessment of PROMs, how frequently was this performed and which tools were used? What is the demography of patients included in RCTs of CCA and which patients report PROMs? What are the reporting standard of studies that include PROMs?	The current standard of PROMs collection and reporting in RCTs of CCA is poor. Greater use of validated PRO measures should better assist clinical decision making	The highest adherence was to the extension item (E15 – report baseline PROs) at 89%. This is a non-specific part of the CONSORT-PRO score, whereas the highest adherence to a specific CONSORT-PRO part was item P1b (identify PRO as a primary or secondary endpoint in the abstract) (62%)	No single study satisfied all the items and there was a wide range of adherence from 1 to 11 out of 14 individual criteria Studies were categorised into high (total scores of 6–11/11), medium (3–5/11) and low (1–2/11) adherence to CONSORT-PRO standards. 14 studies had high adherence scores; another 14 had medium adherence; and 9 had low adherence scores	Ensuring that RCTs include reliable PROMs which matter to patients is an important consideration in delivering value and reducing research waste where important outcomes relevant to users of research are not being assessed
Dos Santos (2017)	This review evaluates the methodological quality of PROs reporting according to the 2013 CONSORT-PROs reporting guidelines in randomized controlled trials (RCTs) evaluating molecular targeted therapies in metastatic renal cell carcinoma (mRCC)	Methodology of PROs assessment needs to be improved with a clear definition of the endpoint and better reporting, monitoring and analyzing to avoid missing data	The most frequently reported items for PRO-specific extension and elaboration were, respectively: the evidence of instrument validity of PROs (55%) and the number of participants included in each analysis required for PROs results (55%). PROs as a primary or secondary outcome in the abstract and/or rationale for the assessment of PROs were described in 41% of publications. However, no publication reported the power of the PROs analysis and only one proposed the impact of the benefits. Most articles (82%) correctly reported the reference of the PROs instrument but no data on the collection methods were reported and only 1/3 described a statistical approach for dealing with missing data. The assessment of PROs at each time point was documented in 50% of the cases and a summary of the results was provided only in 32%. Results from each domain for multidimensional PROs were cited in 41%. In the discussion, half of the publications offered an interpretation of PRO data in relation to clinical outcomes but only 4 (18%) discussed the specific limitations of PROs and the implications for generalizability	The mean score for all items was 4.5 on an 11-point scale (range: 0–11), only 1 publication had a maximum score corresponding to a secondary report	In conclusion, PROs have become an essential endpoint with a strong impact on clinical benefit in patients with mRCC, and they should be increasingly included in RCTs. However, the methodology of PROs assessment needs to be improved with a clear definition of the endpoint that is being used and better reporting with particular attention to monitoring and analyzing in order to avoid missing data
Efficace (2015)	The main objective of this study was to identify the number of RCTs that have included a PRO endpoint across a wide range of cancer specialties and to evaluate completeness of their PRO reporting according to the CONSORT-PRO extension. Secondary objectives were to describe level of reporting by type of PRO endpoint and cancer disease site and investigate which factors are associated with a higher level of reporting	Investigators are encouraged to pay special attention to the CONSORT-PRO items most in need of improvement found in current work to facilitate the use of PRO data to robustly inform patient-centered care in oncology	The two most frequently reported PRO CONSORT items were that of reporting in the abstract that PRO was an outcome of the study (N = 452, 81%) and reporting the use of well-validated PRO instruments (N = 424, 76%). The remaining four items, however, were documented in less than 50% of the RCTs with less than one third reporting a PRO hypothesis (N = 93, 17%), details on statistical approaches for dealing with PRO missing data (N = 113, 20%) and methods for PRO data collection (N = 133, 24%). Overall, less than 5% of RCTs documented all items of the CONSORT-PRO extension. Level of reporting was statistically significant higher in RCTs with PRO as a primary endpoint in four (P1b, P2b, P6aa, P20/21) out of six items (Table 2 of Efficace et al.). A trend toward a greater completeness of reporting for RCTs with PRO as primary endpoint was found. For example, the percentage of RCTs addressing only two items was 58% and 35% for RCTs with PRO as secondary or primary endpoint, respectively	N/R	Also, previous work has shown that editorial policies of journals may vary in how they implement and enforce the original CONSORT statement.23 It is important that future studies will investigate if and how different editorial approaches in endorsing the CONSORT-PRO extension will reflect on accuracy of reporting
Evans (2019)	This study aimed to systematically assess the outcome measures used for measuring PROMs in lateral elbow tendinopathy (LET) in a UK population and to assess the reporting of randomised controlled trials (RCTs) using PROMs in LET	Reporting of outcome measures in lateral elbow tendinopathy RCTs in the UK does not conform to the CONSORT-PRO guidance	Of the 4 studies included in the review, one study identified a PRO as an endpoint in the abstract. The 4 other CONSORT-PRO Extension items were not addressed by any of the 4 RCTs	N/R; however, 3 of the 4 included studies did not address any of the recommended CONSORT-PRO items, scoring 0/5. The 4th study only addressed 1 item	With the increasing use of PROMs used as primary outcomes in clinical trials, it is, therefore, relevant that their use is rigorously assessed
Kyte (2019)	We conducted a systematic evaluation of PRO protocol content and reporting across a cohort of completed international cancer trials	Widespread, non reporting of PRO data means that valuable information is not available for decision making. These deficiencies must be urgently addressed to ensure these data are made available to enhance clinical outcomes for the benefit of future patients	Commonly omitted CONSORT-PRO Extension items included description of the PRO hypothesis/objectives (missing in 71.8% of publications), evidence of the validity and reliability of the PRO instrument(s) (missing in 67.8%), detail regarding the number of PRO data collected at baseline and subsequent time points (missing in 72.8%), and description of the statistical approaches used to deal with missing PRO data (missing in 67.8%)	Where a PRO was the primary outcome, publications included an adjusted mean of 62.1% of CONSORT-2010 items and 41.1% CONSORT-PRO items. Where a PRO was the secondary outcome, protocols included an adjusted mean of 63.3% of CONSORT-2010 items and 16.9% CONSORT-PRO checklist items	That researchers utilize the recently published SPIRIT-PRO Extension [37] alongside the original SPIRIT 2013 statement [28, 42] when developing protocols for trials including PROs. For reporting, the use of the CONSORT-PRO [30] Extension alongside CONSORT[43]. Evidence suggests that the use of such checklists may be valuable in driving up standards of PRO research [44]. Funders and journals should endorse and enforce the use of SPIRIT-PRO and CONSORT-PRO and to promote and facilitate prompt publication of PRO findings, preferably as part of the main trial report. Finally, all stakeholders should utilize the growing range of suitable open access PRO training resources and guidelines to support high-quality PRO research and dissemination
LeBlanc (2020)	The purpose of this review is to summarize what is known about PROs in people living with relapsed/refractory multiple myeloma (RRMM), and to evaluate PRO reporting quality using the CONSORT-PRO Extension guidelines	The format results were reported in made it difficult to describe prevalence, severity or patterns of symptoms and HRQOL issues. Future studies which incorporate PROs would benefit from following existing guidelines to ensure that study evidence and conclusions can be fully assessed by readers, clinicians and policy makers	The most commonly adhered to items from the CONSORT-PRO criteria checklist included ‘PRO data are interpreted in relation to clinical outcomes’ (93%) ‘Assessment timepoints specified’ (90%), ‘PRO identified in abstract as primary or secondary outcome’ (73%) and ‘PRO results for each domain presented’ (73%), (see Table 5 of LeBlanc 2020). The least commonly adhered to items from the checklist included ‘PRO hypothesis stated’ (10%), ‘Method of questionnaire administration specified’ (17%), ‘Statistical approaches for missing data specified’ (23%) and ‘PRO specific limitations and implications for generalizability and clinical practice’	Overall mean reporting quality score was 8.0 out of a possible 15, indicating that on average, manuscripts did not meet 7 CONSORT-PRO criteria. Scores ranged from 2 to 15. Within clinical trials, the highest scores were achieved by manuscripts for which a PRO was identified as a primary endpoint (10.5, n = 10) and for manuscripts that were secondary reports of study results focused on PROs (10.2, n = 10). Mean scores for manuscripts published before the introduction of the CONSORT-PRO guidelines were 7.9 (n = 14) and for manuscripts published after, 8.1 (n = 16)	Future studies which incorporate PROs would benefit from following existing guidelines to ensure that study evidence and conclusions can be fully assessed by readers, clinicians and policy makers
Mack (2018)	The main purpose of this study was to provide evidence examining the adoption of CONSORT-PRO and CERT by researchers examining the link between exercise and quality of life in individuals living with osteoporosis…Guided by [refs [8], [28] of Mack 2018], select study (i.e., study quality) and journal (i.e., impact factor; IF) characteristics were examined to explore their association with CONSORT-PRO scores. Differences between adherence to CONSORT-PRO reporting standards based on whether quality of life served as a primary or secondary outcome and year of publication were also examined	Reporting of PROs in exercise studies for osteoporosis were poor, often not reproducible with inconclusive findings	Authors of primary sources reported "good" evidence for eight (42.1%) CONSORT-PRO items (see Table 2 of Mack 2018). The most frequently reported items were linked to reporting practices found in the Results section of the reviewed studies. Most notably, "good" adherence to reporting standards was noted for the timing of quality of life assessments and the number of participants analyzed at each test administration period (items 10, 11, and 13). Other items coded as "good" were (1) reporting of the PRO in the abstract, (2) reporting baseline scores for quality of life, (3) reporting of PRO results, and (4) reporting clinical outcomes linked to PRO. Nine items (47.37%) were classified as "poor" as less than 50% of the primary sources reported the necessary details. Specifically, mode of administration (n = 2; 8.7%) and details on statistical approaches for dealing with missing PRO data (n = 3; 13%) were rarely evident in the coded studies. For sources with quality of life as a primary endpoint (n = 6), one (4.30%) reported criteria linked to sample size determination. Finally, eight (34.80%) of the primary sources documented information specific to the rationale for including quality of life as an endpoint linked to exercise in individuals living with osteoporosis, included estimates of precision, or identified limitations linked to the PRO	CONSORT-PRO scores across all primary sources ranged from 7.00 to 14.50 (M = 10.17; SD = 1.78). Total scores were not statistically associated with impact factor (r12 = 0.06, p = 0.79) or study quality (r12 = 0.16, p = 0.48). Statistically significant differences in CONSORT-PRO scores when quality of life was the primary (M = 9.58; SD = 1.16) or secondary (M = 10.38; SD = 1.94) endpoint [t(21) = − 0.94, p = 0.36, d = 0.50] were not found. Differences in total scores were not found with publication pre- ( M = 10.44; SD = 1.97) and post (M = 10.29; SD = 1.75) CONSORT-PRO dissemination [t(21) = 0.18, p = 0.86, d = 0.08]	Researchers need to become aware of the recommended methodologies for reporting PRO-related outcomes and those for interventions (TIDieR or CERT) to facilitate critical appraisal and interpretation of the results. Greater use of online supplemental materials now provided by many academic and clinically orientated journals is one avenue to include additional detail. Journal editors, board members, and peer reviewers play an integral role in ensuring that PRO and exercise intervention research are complete and detailed. Journal editors may want to implement select changes that include adherence to the Standards [36]CONSORT-PRO, and CERT to ensure adequacy of reporting within and across studies to advance integrity in published research
Martini (2016)	In detail, this review aims at investigating (i) the amount of available information on HRQoL in patients with Gastroenteropancreatic neuroendocrine tumours, (ii) how HRQoL was assessed and reported, and (iii) if the quality of HRQoL information provided meets agreed standards	Existing HRQOL evidence is hampered by poor methodological quality of existing studies in gastroenteropancreatic neuroendocrine tumours. Future authors should adhere to PRO research guidelines	Instrument validation references and timing of assessments were reported in most studies. PRO hypotheses, mode of instrument administration, statistical power and clinical significance of results was rarely provided. Based on Fig. 2 (of Martini 2016), most studies reported a reference supporting the validity of the instrument and reported rates of missing PRO data. Few studies identified PRO as a primary or secondary endpoint in the abstract, had an a priori PRO hypothesis or reported methods for handling missing PRO data	N/R	Follow PRO guidelines
Mercieca-Bebber (2017) (JPRO)	The aims of this study were to describe the quality of reporting of PROs in ovarian cancer RCTs based on the CONSORT-PRO Extension; describe PRO compliance rates and the reporting of PRO compliance. We also aimed to explore the relationship between CONSORTPRO reporting score and other key variables which we thought may influence reporting, including whether there was a significant difference in the primary trial endpoint or the PRO endpoint, compliance rates and year of publication. We also explored whether the PRO content of the ovarian cancer RCT protocols reviewed previously [25] had an impact on: (1) the overall standard of PRO reporting according to the CONSORT-PRO, and (2) PRO compliance	PRO reporting is in need of improvement, particularly with regard to reporting rates, reasons and handling of missing PRO data. Use of SPIRIT-PRO and CONSORT-PRO will ensure high-quality PRO findings are accurately interpreted and can meaningfully impact patient care	27 (75%) RCTs reported results of pre-specified PRO endpoints or all domains of the PRO questionnaire used, 25 (69%) interpreted PROs in the context of clinical endpoints, 19 (53%) provided the number of participants included in each PRO analysis, and 23 (64%) cited evidence of the validity of the PRO questionnaire used. However, other items were reported poorly; most concerning was the limited number of RCTs reporting baseline PROs (n = 13, 36%), or reporting approaches for dealing with missing PRO data (n = 14, 39%)	Total CONSORT-PRO scores (n = 36) ranged from 0 to 13.5/14, with a mean of 6.7 (48%). Most (n = 33, 92%) reported some PRO results. Of the 3 (12%) RCTs that did not report any PRO results, 2 stated that these would be reported subsequently (CONSORT-PRO total scores of 0/14 and 1/14, respectively). The other did not analyse the PRO data due to poor compliance, and did not address any other recommended CONSORT-PRO criteria, scoring 0/14. Another low-scoring publication (scoring 1/14) simply reported that there were no differences in global QOL at any time point, but did not report the time points assessed, analysis methods, or results for other questionnaire domains	PRO studies must be designed, conducted and reported to the highest standards to be of most benefit to patient care. Our findings suggest that: (1) adherence to the forthcoming SPIRIT-PRO Extension and CONSORT-PRO Extension for the development of protocols and publications, respectively, and (2) prospectively collecting reasons for missing data and reporting these reasons in the publication, can assist researchers to ensure that high-quality PRO evidence is available and utilised in clinical practice
Mercieca-Bebber (2017) (QOLR)	We sought to (1) assess the uptake of CONSORT-PRO by identifying articles that cited the CONSORT-PRO Extension in the first 3 years since its release; (2) identify published RCTs that cited CONSORT-PRO and describe their adherence to the statement; (3) compare the quality of PRO reporting in RCTs that cited CONSORT-PRO to a control sample; (4) identify predictors of CONSORT-PRO adherence; (5) identify which journals publish RCTs with PRO endpoints, so that these journals can be included in future knowledge transfer efforts led by the ISOQOL Reporting Taskforce and (6) describe to what extent journals publishing RCTs with PRO endpoints endorse CONSORT-PRO	Reporting of the PRO endpoint in a dedicated publication, journal endorsement of CONSORT-PRO and citing CONSORT-PRO were significant predictors of higher total CONSORT-PRO adherence scores. Many key journals do not endorse CONSORT-PRO in their instructions to authors	Cases (citing CONSORT-PRO) frequently reported a rationale for including PROs, evidence of instrument validity, who completed the measure, questionnaires available at principle timepoint for analysis, results for hypothesised domains, implications for generalisability and interpreted PRO results with clinical outcomes. Cases rarely reported the mode of questionnaire administration. Controls (articles not citing CONSORT—PRO) frequently reported a rationale for PROs, baseline PRO scores, generalisability and interpreted PROs with clinical items. Controls rarely identified PROs in the abstract, PRO hypotheses, mode of administration or results of exploratory analyses	The 26 cases (RCTs citing CONSORT-PRO) had significantly higher total CONSORT-PRO adherence scores (mean 77.7% of items, range 46.7–100%), compared to controls (comparable RCTs not citing CONSORT-PRO) mean 67.6%, range: 25.0–96.4%), t = 2.64, p = 0.01. For the extension adherence score (5 Extension items only), a larger difference was found between cases (mean 77.5%, range 28.6–100%) and controls (mean 59.5%, range 21.4–92.9%), t = 4.50, p < 0.001 There were three significant predictors of higher CONSORT- PRO total adherence score: ‘citing CONSORTPRO’, ‘journal endorsing CONSORT-PRO’ and ‘dedicated PRO paper’ (R2 = 0.48, p < 0.001). In the model for the five extension items only, there were two significant predictors: ‘citing CONSORT-PRO’ and ‘journal endorsing CONSORT- PRO’ (R2 = 0.36, p < 0.001). We did not observe a relationship between the year of publication and CONSORT-PRO total adherence score (r = 0.11, p = 0.39) or Extension adherence score (r = 0.05, p = 0.68)	Journals should endorse EQUATOR guidelines. Key primary results should be reported with the primary trial publication, detailed analyses should be reported in a dedicated QOL publication for the trial
Stevens (2016)	The aim of this study was to summarize current evidence regarding the collection of PRO data in RCTs of unplanned general surgery and to use this information to inform the design of future studies in this area	There is a lack of well designed, and conducted RCTs in unplanned general surgery that include PROs	Best reported: PRO data interpreted alongside clinical outcomes was completed for 27 of 35 PROs (74.3%) across the 20 studies and Reporting of the person completing the PRO for 19 PROs (54.3%). Worst reported items: PRO hypothesis stated in background /objectives 5 (14.3%), PROs used in eligibility/stratification criteria 0, Explicit statement of statistical approaches for dealing with missing data 1 (2.9%), Reporting of number of patients completing PROMs at follow-up 13 of 99 FU timepoints (13.1%), Additional analyses reported, included distinction between pre-specified and exploratory 0	N/R	More intervention research needed; use of core outcome set
Van Der Weijst (2019)	In addition, the methodological quality of this set of papers is analyzed with the ultimate goal to discuss challenges in and recommendations for the interpretation and comparison of HRQoL evidence obtained from randomized controlled trials (RCTs)	Poor reporting and heterogeneity of methods makes comparisons of HRQOL outcomes hardly feasible HRQoL outcomes remains poor with certain aspects being systematically underreported	All PRO items were scored poorly, except for identification PRO as a primary or secondary outcome in the abstract which was scored moderate. Only one study fulfilled all criteria…60% of studies identified PROs in the abstract, however PRO hypotheses were available for only 13% of studies, instrument validity in 6% of studies, approaches for missing data in 18% of studies and interpretation with clinical outcomes in 31% of studies	N/R	Future clinical trials exploring novel therapies for advanced NSCLC should focus on reporting HRQoL data in a clinically meaningful and methodologically qualitative way. Additionally, further research should focus on developing standards to optimize and on defining MCID scores
Weingartner (2016)	The aim of this review was to assess whether and how PROs are measured and reported in publications of RCTs evaluating anti-cancer therapies in advanced cancer according to the Consolidated Standards of Reporting Trials (CONSORT) PRO extension	The assessment, reporting and discussion of relevant PRO should be mandatory in the primary publication of trials that enroll patients with advanced and life-threatening diseases, to assist clinical decision-making in advanced cancers	25(83%) of the 30 publications that reported PRO results verified the ‘evidence of the PRO instrument’ (item P6a). Also ‘outcomes and estimation’ (item 17, 67%) and ‘number analyzed’ (item 16, 60%) were usually met. 16 publications (53%) interpreted the results of the PRO data ‘in relation to clinical outcomes, including survival data’ (item 14). Nine publications (30%) mentioned the PRO as study outcome in the abstract (item P1b). The ‘stated PRO hypothesis’ (item P2b, 13%) and ‘ancillary analyses’ (item 18, 7%) were rarely mentioned. Item 7 (sample size) was not met by any publication	Mean (SD) 4.4 (2.5) of 14 items were met	Adequate assessment and reporting of PRO should become mandatory in the primary publication of trials that enroll patients with advanced and life-threatening disease PRO reporting and discussion need to be further advocated, so that it may lead to an understanding of the patient experience that may inform decisions by patients, providers, regulators, and payers better and enables an informed and patient-oriented decision making Specifically, if HRQoL and symptom burden would be assessed and reported adequately and considered together with the results for overall survival, patients, physicians, health care authorities and decision-makers will be provided with all necessary information to weight patient benefit versus potential patient burden in this critical and vulnerable phase of life

One study assessed predictors of higher adherence to CONSORT-PRO items, and determined that citing CONSORT-PRO, publishing in a ‘journal endorsing CONSORT-PRO’ and ‘publishing PROs in a dedicated PRO paper’ were predictors of higher CONSORT-PRO adherence scores [17]. Other studies also noted a trend for higher adherence scores for articles that reported PROs in a dedicated, secondary publication [15, 26, 28].

### Journal endorsement of CONSORT-PRO

Of the 13 journals that the 14 included review articles were published in, none recommended use of the CONSORT-PRO Extension specifically, five recommended use of EQUATOR guidelines or appropriate CONSORT guidelines, and nine journals did not mention either in their instructions to authors.

## Discussion

Our review found that evaluation checklists developed based on the CONSORT-PRO recommendations are inconsistent and lead to differing evaluations of reporting of RCTs. The key features of the CONSORT-PRO recommendations that have been adapted for these evaluations include: the number of CONSORT-PRO Extension items included, the number of CONSORT-PRO Elaboration items included, the weighting of specific items—particularly those that address multiple aspects of reporting, the wording of certain items and the highest possible total score that could be obtained from evaluation.

As noted earlier, these review articles are, by their nature, sending a message to readers about how PROs should be reported. Readers of these review articles may incorrectly assume that if a CONSORT-PRO item is missing from the evaluation criteria of a specific review, then it is not essential to include that item in future reports. This issue is made even more problematic when articles make judgements about what constitutes a “good” total CONSORT-PRO reporting score. One article, with a highest possible total CONSORT-PRO adherence score of 11, suggested that studies scoring between 6 and 11/11 had high adherence, studies scoring between 3 and 5/11 had medium adherence, and between 1 and 2/11 had low adherence to CONSORT-PRO standards—without accounting for CONSORT-PRO recommendations for articles with a primary PRO endpoint only. As with any reporting standards endorsed by the EQUATOR network, in the interests of promoting high-quality, transparent reporting and reducing waste, all CONSORT-PRO items are recommended for inclusion in publications of PRO trial endpoints, unless specific conditions are specified for individual items. For example, CONSORT-PRO recommends a PRO-specific sample size calculation only for studies with a primary PRO endpoint, so a specific calculation for PROs is not required where PROs are secondary endpoints. The aim of these resources is not to criticise researchers, but to help researchers to publish high-quality and clear reports of their research, so that stakeholders may interpret the findings accurately and use the results appropriately in clinical practice or policy decisions. The implication of not reporting research clearly or completely is that readers may misunderstand the purpose and value of the research, the methods and/or findings. Readers may assume that PRO data does not exist for particular important clinical questions. This may limit results from impacting clinical practice, or result in duplication of research efforts in the future (researchers may assume those research questions need answering in future studies), which may be viewed as a waste of research resources and participant time.

We noted differences in reporting practices between reviews in the CONSORT-PRO items most- and least-often reported. All 14 review articles reported that the statistical approach used for handling missing PRO data (item P12a) was poorly reported, including articles published before and after the CONSORT-PRO was released. This item, in combination with item E16 (number of participants included in each analysis), is of particular importance to help readers judge the potential bias resulting from missing PRO data, and the extent to which results can be generalised to the broader patient population. Numerous publications over the past 30 years have highlighted this reporting issue and offered suggestions for how to address it [31–42].

A related issue highlighted by many of the review articles was that many studies did not report results for each assessed domain and assessment time point (E17a). This item was omitted from the evaluation of five of the 14 review articles. In PRO research, multi-domain instruments are often used, yet often only the overall quality of life domain is reported. This may contribute to research waste because readers may assume that other domains were not collected and that research questions around these domains remain unanswered. Given that leading regulators are advocating for the assessment of specific and clearly defined PRO domains that are more proximal to the studied disease or condition [43], there is a clear need for improved identification and definition of PRO endpoints, in addition to specification of their primary or secondary endpoint status. Calvert et al. [11] noted the importance of full reporting of all domains assessed to avoid bias in reporting, in justifying the need for and purpose of this item in the CONSORT-PRO checklist. Options to overcome challenges associated with restrictive manuscript word limits include publishing PROs in a dedicated publication, or including detailed PRO results in a manuscript appendix.

We also noted differences in the recommendations between review articles, which in some cases contradicted one another. The original CONSORT-PRO article [11] recommends that key PRO endpoints are published in the main trial publication, however review studies conducted since have observed that PRO reporting is more complete when PROs are published in a dedicated publication [15, 17, 26, 28]. Accordingly, these reviews have recommended that future trials publish their PRO data in a separate publication as soon as possible after publishing their primary trial endpoints. Two key details to this approach being successful, and to minimise the potential for research waste, are to clearly report trial identifying details (e.g., trial registration number, trial name) across all trial publications (cross-referencing where possible), and to ensure all endpoints are published in a timely manner, as there is a risk that certain endpoints will be left unpublished if not included in a single publication.

Our results highlight the need for training across health and medical setting in all aspects of PRO reporting, to encourage effective use of CONSORT-PRO, with the outcome of reducing research waste. A new initiative, patient-reported outcomes tools: engaging users and stakeholders (PROTEUS) aims to advance the use of PROs in research studies and clinical practice by implementing and disseminating CONSORT-PRO and related resources. The PROTEUS website includes checklists, web tutorials, and other resources to support the optimal use of PROs so that patients, clinicians, and other decision-makers have the information they need to support patient-centered care [44]. Education about both the *importance* of high-quality research design, conduct and reporting, and *how* to implement these through use of available resources, is crucial to minimise the risk of PRO research waste in the future.

### Recommendations

As noted earlier, CONSORT-PRO was not intended to be used as an evaluation tool, however at least 14 studies have used the guidance in this way since its publication. Whilst these studies have helped to highlight what areas of PRO reporting are in need of improvement generally (despite the existence of guidance), studies using incomplete or modified checklists may also have confused readers about what is recommended in a publication of PRO trial endpoints, which may in turn result in incomplete or poor quality reporting and consequential research waste. We recommend that future studies using the CONSORT-PRO guidance to evaluate studies take care to include all CONSORT-PRO items (elaborations and extensions). We acknowledge that it is difficult to assess multi-component outcomes of the CONSORT-PRO checklist, and it is worth taking a consistent approach to future evaluations by adapting the CONSORT-PRO extension for review purposes so that all components are clearly assessable. We have attached a template (Appendix 2) that addresses all component items, which future reviews may wish to implement. This checklist has been used successfully in three of the included review studies [17, 23, 25].

Future review articles should also encourage readers to seek high-quality PRO advice and support when planning and publishing PRO studies, to ensure that reporting is clear, accurate and in line with recommended standards. This practice would facilitate appropriate knowledge translation of the CONSORT-PRO extension. The PROTEUS website may be useful for this purpose. It covers all aspects of PRO design, methodology, analysis, interpretation and reporting, and draws on expert, internationally endorsed PRO guidance in its content. Research manuscripts are a key source of information for policy makers, funders, clinicians, patients and their families, when making important treatment and clinical decisions, therefore we should strive to produce and communicate data of the highest quality. Glasziou and colleagues have outlined strategies to reduce research waste resulting from poor reporting which apply to PROs, including following EQUATOR reporting guidelines; registering trials, using CrossRef to link reports to trials; making use of archiving software to facilitate data sharing, data back up and collaboration; producing commentaries post-publication; and ensuring findings are presented within research context [3].

### Strengths and limitations

We used a robust search strategy across multiple databases to identify suitable manuscripts for this review, and two experienced reviewers double screened all abstracts. We also followed the PRISMA guidance in designing, conducting and reporting our review. However, it is possible that human errors may have led to relevant articles being missed. Our review only identified papers written in English. It is possible that if we had searched international databases, relevant non-English articles may have been identified, particularly as CONSORT-PRO has recently been translated into Japanese [45]. We also acknowledge that it is only fair to describe, not evaluate, RCT publications published before CONSORT-PRO was released in 2013 using CONSORT-PRO, however all reviews included in this review of reviews, included at least one paper published before CONSORT-PRO was released. We included all review studies that met our inclusion criteria and purposefully did not assess their methodological quality. We wanted to focus on the specific CONSORT-PRO-inspired reporting evaluation checklists used, and how these may impact future reporting, rather than the methodological quality of the systematic reviews themselves, as arguably the review checklists used would impact knowledge translation more directly. The completeness of the CONSORT-PRO-inspired checklist is most relevant to our study’s aim, and in the absence of any measures of effect, an additional assessment of methodological quality is unlikely to impact our descriptive findings [46]. As noted above, we found that some evaluation checklists were incomplete, therefore, our summary of item-level reporting of CONSORT-PRO criteria are limited accordingly.

We did not check for overlap between the 1181 studies evaluated across the 14 included reviews. There is potential for overlap, particularly as eight of the reviews were specific to oncology, and two of the eight studies (*N* = 681 studies) included any cancer diagnosis. The remaining six reviews of oncology RCTs looked at reporting in specific cancer populations. Some of these studies did not publish lists of included studies, therefore we could not check the extent of overlap. Cochrane’s guidance on conducting reviews of reviews suggests that some degree of overlap in descriptive reviews such as ours is not problematic [46].

Three of the reviews included a small number of studies which were not RCTs [23, 24, 28], for which the CONSORT-PRO guidance may be less relevant.

Finally, we wish to reiterate that adherence to reporting guidelines represents only one strategy to reduce research waste and promote research impact. There is no measurable, linear relationship between adherence to the CONSORT-PRO checklist and the extent of research waste, as research impact is a complicated and multi-faceted concept. A poorly designed and conducted study can indeed report every item on the CONSORT-PRO checklist, and a study that is designed and conducted perfectly may not address all items on the CONSORT-PRO checklist in its publication. A paper that adheres to reporting guidelines better-places a reader to assess its design and conduct and to interpret its findings accurately—consequently improving the potential of the research to be impactful and meaningful to clinical practice, and to contribute to systematic reviews or meta-analyses.

## Conclusions

Our study has two major findings. Firstly, many PRO studies published after 2013 (when CONSORT-PRO was published) did not report items recommended by CONSORT-PRO, which may lead to research waste if results are consequently misinterpreted or misunderstood. Additional knowledge translation efforts are needed to promote effective use of the CONSORT-PRO guidance. Secondly, studies reviewing PRO publications have omitted or largely modified recommended items from their evaluations, which may cause confusion and further issues for readers regarding how best to report their PRO research in line with the CONSORT-PRO extension.

When reported effectively, PRO data from RCTs can provide valuable information to inform clinical care, regulatory decision-making and health policy. The CONSORT-PRO extension aims to improve the completeness  and transparency of reporting, to maximise research impact and help minimise research waste. Articles reviewing PRO reporting have successfully identified areas in need of improvement, but may also have caused readers to believe that certain CONSORT-PRO items are non-essential or optional due to omitting these items from their evaluations, which is not the case. Future review articles should include all 14 CONSORT-PRO Extensions and Elaborations to avoid confusion or issues with knowledge translation, and should recommend readers access helpful resources to promote high-quality PRO reporting.

## Supplementary Information

Below is the link to the electronic supplementary material.

Supplementary file1 (DOCX 18 KB)

Supplementary file2 (DOCX 15 KB)

## Declarations

### Research involving human and animal participants

This article does not contain any studies with human participants or animals performed by any of the authors.
