# Supplementary material for: Knowledge translation concerns for the CONSORT-PRO extension reporting guidance: a review of reviews
Source: Qual Life Res. 2022 Mar 26;31(10):2939–57. doi: 10.1007/s11136-022-03119-w (PMC9470606; doi:10.1007/s11136-022-03119-w)
Supplement: Supplementary file 1 — Supplementary file1 (DOCX 18 KB) [file 11136_2022_3119_MOESM1_ESM.docx]

Appendix 1. List of included studies

1. Bylicki O, Gan HK, Joly F, Maillet D, You B, Peron J. Poor patient-reported outcomes reporting according to CONSORT guidelines in randomized clinical trials evaluating systemic cancer therapy. Ann Oncol. 2015;26(1):231-7.

2. Chen Y, Nagendran M, Gomes M, Wharton PV, Raine R, Lambiase PD. Gaps in patient-reported outcome measures in randomized clinical trials of cardiac catheter ablation: a systematic review. Eur Heart J Qual Care Clin Outcomes. 2020;6(4):234-42.

3. Dos Santos M, Brachet PE, Chevreau C, Joly F. Impact of targeted therapies in metastatic renal cell carcinoma on patient-reported outcomes: Methodology of clinical trials and clinical benefit. Cancer Treat Rev. 2017;53:53-60.

4. Efficace F, Fayers P, Pusic A, Cemal Y, Yanagawa J, Jacobs M, et al. Quality of patient-reported outcome reporting across cancer randomized controlled trials according to the CONSORT patient-reported outcome extension: A pooled analysis of 557 trials. Cancer. 2015;121(18):3335-42.

5. Evans JP, Smith C, Porter I, Gangannagaripalli J, Goodwin V, Valderas J. Patient-centred outcomes in lateral elbow tendinopathy: A systematic review of available evidence in UK populations. Shoulder Elbow. 2019;11(6):440-9.

6. Kyte D, Retzer A, Ahmed K, Keeley T, Armes J, Brown JM, et al. Systematic Evaluation of Patient-Reported Outcome Protocol Content and Reporting in Cancer Trials. J Natl Cancer Inst. 2019;111(11):1170-8.

7. LeBlanc MR, Hirschey R, Leak Bryant A, LeBlanc TW, Smith SK. How are patient-reported outcomes and symptoms being measured in adults with relapsed/refractory multiple myeloma? A systematic review. Qual Life Res. 2020;29(6):1419-31.

8. Mack DE, Wilson PM, Santos E, Brooks K. Standards of reporting: the use of CONSORT PRO and CERT in individuals living with osteoporosis. Osteoporos Int. 2018;29(2):305-13.

9. Martini C, Gamper EM, Wintner L, Nilica B, Sperner-Unterweger B, Holzner B, et al. Systematic review reveals lack of quality in reporting health-related quality of life in patients with gastroenteropancreatic neuroendocrine tumours. Health Qual Life Outcomes. 2016;14(1):127.

10. Mercieca-Bebber R, Friedlander M, Calvert M, Stockler M, Kyte D, Kok PS, et al. A systematic evaluation of compliance and reporting of patient-reported outcome endpoints in ovarian cancer randomised controlled trials: implications for generalisability and clinical practice. JPRO. 2017;1(1):5.

11. Mercieca-Bebber R, Rouette J, Calvert M, King MT, McLeod L, Holch P, et al. Preliminary evidence on the uptake, use and benefits of the CONSORT-PRO extension. Qual Life Res. 2017;26(6):1427-37.

12. Stevens DJ, Blencowe NS, McElnay PJ, Macefield RC, Savovic J, Avery KN, et al. A Systematic Review of Patient-reported Outcomes in Randomized Controlled Trials of Unplanned General Surgery. World J Surg. 2016;40(2):267-76.

13. Van Der Weijst L, Lievens Y, Schrauwen W, Surmont V. Health-Related Quality of Life in Advanced Non-small Cell Lung Cancer: A Methodological Appraisal Based on a Systematic Literature Review. Front. 2019;9:715.

14. Weingartner V, Dargatz N, Weber C, Mueller D, Stock S, Voltz R, et al. Patient reported outcomes in randomized controlled cancer trials in advanced disease: a structured literature review. Expert Rev Clin Pharmacol. 2016;9(6):821-9.

List of studies EXCLUDED at the full text stage, with reasons for exclusion

|  | **Article excluded at full text screening stage** | **Reason for exclusion** |
| --- | --- | --- |
|  | Gianola S, Frigerio P, Agostini M, Bolotta R, Castellini G, Corbetta D, et al. Completeness of Outcomes Description Reported in Low Back Pain Rehabilitation Interventions: A Survey of 185 Randomized Trials. Physiotherapy Canada. 2016;68(3):267-74. | Review used checklist other than CONSORT-PRO |
|  | Gilbert A, Ziegler L, Martland M, Davidson S, Efficace F, Sebag-Montefiore D, et al. Systematic Review of Radiation Therapy Toxicity Reporting in Randomized Controlled Trials of Rectal Cancer: A Comparison of Patient-Reported Outcomes and Clinician Toxicity Reporting. Int J Radiat Oncol Biol Phys. 2015;92(3):555-67. | Review used checklist other than CONSORT-PRO |
|  | Hamaker ME, Schulkes KJ, Ten Bokkel Huinink D, van Munster BC, van Huis LH, van den Bos F. Evaluation and reporting of quality of life outcomes in phase III chemotherapy trials for poor prognosis malignancies. Qual Life Res. 2017;26(1):65-71. | Not review of reporting |
|  | Patrick D. Reporting of patient-reported outcomes in randomized trials: the CONSORT PRO extension. Value Health. 2013;16(4):455-6. | Not review of reporting |
|  | Ter Veer E, van Kleef JJ, Sprangers MAG, Haj Mohammad N, van Oijen MGH, van Laarhoven HWM. Reporting of health-related quality of life in randomized controlled trials involving palliative systemic therapy for esophagogastric cancer: a systematic review. Gastric Cancer. 2018;21(2):183-95. | Review used checklist other than CONSORT-PRO |
|  | Van Lieshout EMM, Wijffels MME. Patient-reported outcomes: Which ones are most relevant? Injury. 2020;51 Suppl 2:S37-S42. | Not review of reporting |
|  | Chakraborty R, Cannella L, Cottone F, Efficace F. Quality of patient-reported outcome reporting in randomised controlled trials of haematological malignancies according to international quality standards: a systematic review. Lancet Haematol. 2020;7(12):e892-e901. | Review used checklist other than CONSORT-PRO |
|  | Mouillet G, Efficace F, Thiery-Vuillemin A, Charton E, Van Hemelrijck M, Sparano F, et al. Investigating the impact of open label design on patient-reported outcome results in prostate cancer randomized controlled trials. Cancer Med. 2020;9(20):7363-74. | Review used checklist other than CONSORT-PRO |
