# Supplementary material for: Knowledge translation concerns for the CONSORT-PRO extension reporting guidance: a review of reviews
Source: Qual Life Res. 2022 Mar 26;31(10):2939–57. doi: 10.1007/s11136-022-03119-w (PMC9470606; doi:10.1007/s11136-022-03119-w)
Supplement: Supplementary file 2 — Supplementary file2 (DOCX 15 KB) [file 11136_2022_3119_MOESM2_ESM.docx]

**Appendix 2. CONSORT-PRO review criteria & scoring**

Adapted from: Calvert M, Blazeby J, Altman DG, et al. Reporting of patient-reported outcomes in randomized trials: The CONSORT-PRO extension. JAMA 2013; 309: 814-822.

**P1b. Abstract – PRO noted as primary/secondary endpoint**

(1= yes, 0.5= PRO mentioned but unclear endpoint status; 0=no)

**2a. Background and rationale for including PROs**

(1= yes, 0=no)

**P2bi. PRO hypothesis present**

(0.5= yes, 0=no)

**P2bii. PRO domains specified in hypothesis**

(0.5= yes, 0=no)

**4a. PRO eligibility or stratification criteria (if used)**

(1= yes, 0=no)

**P6ai. Evidence of PRO instrument validity provided/cited**

(1= yes, 0=no)

**P6aii. Statement of the person completing the PRO measure (e.g. 'patients completed', or 'self-report')**

(0.5= yes, 0=no)

**P6aiii. Mode of administration specified (e.g. paper, e-PRO)**

(0.5= yes, 0=no)

**7a. (Primary PRO endpoint only) how sample size was determined**

(1= yes, 0=no)

**P12a. Statistical approach for dealing with missing data specified (e.g. imputation, omission of cases with missing data)**

(1= yes, 0=no)

**13ai. Report number of questionnaires submitted/available for analysis at baseline**

(0.5= yes, 0=no)

**13aii. Report number of questionnaires submitted/available for analysis principle timepoint for PRO analysis**

(0.5= yes, 0=no)

**15. Demographics table includes baseline PRO findings**

(1= yes, 0=no)

**16. Number of patients (denominator) included in each PRO analysis and whether this was intention to treat**

(1= yes, 0=no)

**17ai. PRO results reported for the hypothesised domains and time point specified in the hypothesis –OR- reported for each domain of the PROM if no PRO hypothesis provided**

(0.5= yes, 0=no)

**17aii. Results include confidence intervals, effect size or some other estimate of precision**

(0.5= yes, 0=no)

**18. Results of any subgroup/adjusted/exploratory analyses are reported**

(1=yes, 0=no)

**P20. PRO study limitations provided**

(1=yes, 0=no)

**P21. Implications of PRO results for generalizability, use in clinical practice**

(1=yes, 0=no)

**22. PROs interpreted in relation to clinical outcomes**

(1=yes, 0=no)
